# Bee Venom Melittin Modulates In Vivo Water Permeability of Red Blood Cells: Microscopic and ^1^H-NMR Data

**DOI:** 10.3390/molecules30224419

**Published:** 2025-11-15

**Authors:** Ștefana Bâlici, Adrian Florea, Ameen Ibrahim Al-Hajaj, Alin-Dan Chiorean, Gheorghe Zsolt Nicula

**Affiliations:** Department of Cell and Molecular Biology, Faculty of Medicine, “Iuliu Hațieganu” University of Medicine and Pharmacy, 6 Louis Pasteur St., 400349 Cluj-Napoca, Romania; sbalici@umfcluj.ro (Ș.B.); gnicula@umfcluj.ro (G.Z.N.)

**Keywords:** melittin, bee venom, red blood cells, membranes, ^1^H-NMR spectroscopy, water permeability, aquaporin

## Abstract

Bee venom (BV) molecules, including melittin (Mlt), are known to modify the permeability of membranes. This paper assessed red blood cell (RBC) shape (by phase contrast microscopy) in relation to some of the parameters (haematology data) and calculated the RBC membranes’ water diffusional permeability (P_d_) with ^1^H-NMR spectroscopy. Rats were injected for 30 days with either small daily doses of BV (VST) or Mlt (MST) or with high single doses of BV (VSLT) or Mlt (MSLT). The RBCs displayed aberrant shapes, all of the analysed parameters significantly changed, and the values of P_d_ were higher (and increased with temperature) in all of the treated groups compared to the control group. The RBCs in the venom-treated groups had the highest mean values (expressed in cm × s^−1^ × 10^3^) of P_d_ at 37 °C—8.95 in the VSLT group and 8.69 in the VST group—which were followed by the MST and MSLT groups and the control group. Our results demonstrated the ability of Mlt to retain the ability to interact with the RBC membrane in vivo and proved that Mlt is the most important BV molecule involved in this process.

## 1. Introduction

Water, an essential molecule for life, is required for most of the extracellular and intracellular metabolic processes in organisms. This is why cellular membranes exhibit a physiological permeability for water, which is either unspecific or achieved by a transport protein first identified in the red blood cell (RBC) membrane [1,2] and later called CHIP28 [3] or aquaporin (AQP) [4,5,6]. The transport of water by AQPs across the cell membranes of certain organs or tissues explains their various specific functions or pathologies [7,8,9,10]. AQP1 is responsible for the shape and volume of RBCs and also for exchanging water between RBCs and various tissues [11]; recent studies implicated this molecule in the transport of oxygen across the RBC membrane in certain conditions [12] and in oxidative stress [13].

The water permeability of membranes can be modulated by different external physical or chemical factors, leading to changes in the normal functions of cells. Among chemical factors, small, cationic peptides are of interest for this paper. These are molecules with fewer than 50 amino acids, with low molecular weight and with net positive electric charge [14,15], and they are useful tools for the study of membranes. In nature, such molecules are synthesised by a wide range of organisms as part of their defences against bacteria [16]. Cationic peptides have been extensively tested as antimicrobial and antifungal agents, which is a very important aspect in the context of increased bacterial resistance against commonly used antibiotics [17].

Honeybees (*Apis mellifera*) have developed a high-level biochemical pharmacology during their evolution. Bees synthesise several biologically active products, and bee venom (BV) is distinguished by its great complexity. Habermann showed that BV consists of numerous organic and inorganic substances [18]. Among the former, melittin (Mlt) prevails in the composition of BV [18] and in terms of effects as well [19]. Mlt is a typical cationic peptide and represents up to 50–55% of the venom dry weight. The molecular weight of Mlt separated by electrophoresis was around 12,000 Da [18,20], corresponding to a tetramer with identical linear chains of 26 amino acids. The Mlt monomer molecular weight is 2846.53 Da [21,22,23]. Mlt is an amphiphilic peptide [18] with a hydrophobic N-terminus (starting with amino acid residue 20), while the C-terminal residues (up to the sixth) are strongly basic and hydrophilic [24]. Due to its properties, Mlt has high affinity for biological membranes. The presence of Mlt in large doses in the RBC membrane results in the alteration of normal water exchange, culminating with haemolysis as an important pathological feature of BV envenomation. This is also a common effect produced by the venoms of other invertebrate [25,26,27,28] and vertebrate [29,30,31] animals via different mechanisms. The fragmentation or even destruction of RBCs by BV toxins is often followed by severe complications, acute renal failure, or even death associated with important haematological changes [32,33,34]. The medical importance of BV envenomation is particularly relevant in cases of multiple bee stings, which is a growing public health concern [35,36,37,38]. In the literature, there are several reports of the in vitro effects of RBCs incubation with venom molecules. Zalat et al. [39] studied the haemolytic effect provided by BV and analysed some haematological parameters. Another study showed the ability of different venoms to induce changes in the RBC shapes with the authors reporting crenated cells after incubation with BV [40]. Also, data based on interferential contrast microscopy, confocal microscopy, and NMR measurements showed changes in RBC membrane morphology associated with substantially reduced anion-exchange activity under the influence of four snake venoms [41]. The study by Yau et al. [41] is also the only one found by us in which the RBC membrane parameters were assessed with NMR methodology.

Although the interaction of the BV molecules with membranes was extensively studied, there are no in vivo studies that establish a relationship between their presence in the natural environment of the RBCs and certain possible shape alterations of these cells. Moreover, the permeation of the membranes by Mlt is well documented and commonly accepted, but there are no in vivo studies that investigate potential changes in the water permeability of the RBC membranes in the presence of the BV molecules, including Mlt. It is also not known whether Mlt insertion in the RBC membranes affects somehow their functions by perturbing the normal rate of water transport. In this context, we aimed to assess for the first time the ability of BV, and of Mlt, alone to trigger in vivo morphological reactions of RBCs consequently to their subcutaneous administration in rats in different experimental conditions. The second and the main objective of the study was to first precisely calculate the membrane permeability for water with ^1^H-NMR spectroscopy in RBCs collected from rats after the in vivo experimental administration of BV and Mlt and then establish a correlation between the values of this parameter and the morphological effects.

## 2. Results

### 2.1. RBCs Morphology

#### 2.1.1. Control Group

**Figure 1:**
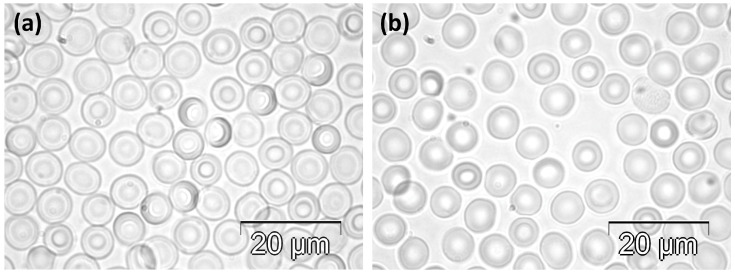
Phase contrast microscopy images illustrating the normal morphology of RBCs in rats in the C group. (**a**) Higher and (**b**) lower density of cells.

RBCs collected from the rats in the C group were biconcave discs, as presented in Figure 1. Rare cells showed slightly irregular contours (Figure 1a,b), and oval shapes were even rarer (Figure 1b).

#### 2.1.2. Bee Venom Treated Groups

**Figure 2:**
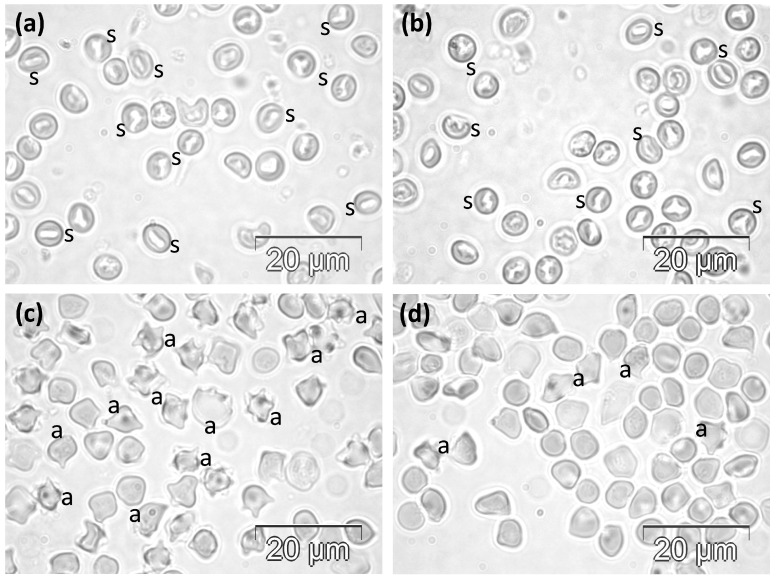
Phase contrast microscopy images showing the abnormal morphology of RBCs in rats in (**a**,**b**) the VST group and (**c**,**d**) the VSLT group. a, acanthocytes; s, stomatocytes.

The subchronic experimental treatment with daily doses of 700 μg BV/kg/day was followed by important morphological changes in the RBCs (Figure 2a,b). The RBCs were visibly smaller than those in the C group, and a significant number of cells with aberrant shapes were observed as well. They were mostly stomatocytes, but some intermediate shapes were present as well. Rare cells had a borderline aspect between stomatocytes and acanthocytes (Figure 2a).

The acute semi-lethal experimental treatment with single doses of 62 mg BV/kg significantly affected the cellular morphology, with almost all RBCs showing specific features of acanthocytes, with smaller or longer projections (Figure 2c,d). Only very few cells preserved the normal shape of biconcave disc, while some others had more or less irregular contours.

#### 2.1.3. Melittin-Treated Groups

**Figure 3:**
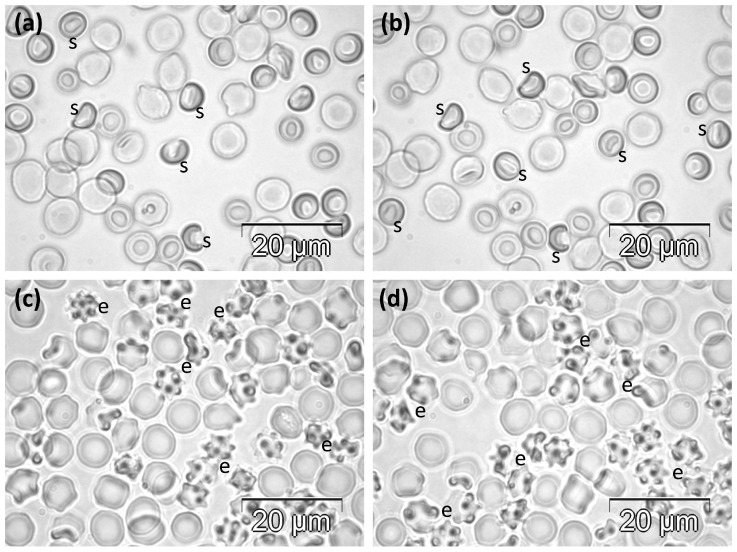
Phase contrast microscopy images showing the abnormal morphology of RBCs in rats in (**a**,**b**) the MST group and (**c**,**d**) the MSLT group. e, echinocytes; s, stomatocytes.

After the experimental treatment for 30 days with daily doses of 350 μg Mlt/kg, many RBCs displayed various sizes and abnormal shapes. They mainly showed irregular contours, and some of them were stomatocytes. However, some cells still preserved their normality (Figure 3a,b).

The experimental treatment with the high, single doses of 31 mg Mlt/kg resulted in morphological changes affecting more than half of all cells. Most of them were echinocytes of different degrees. A low number of RBCs remained discocytes, while some others showed irregular contours (Figure 3c,d).

### 2.2. RBC Diameters

The RBC diameters in the five experimental groups are presented in Table 1. The mean RBC diameter in the VST group was the lowest among of the all experimental groups, which had statistical significance when compared to those in the C, MST, and MSLT groups (Table 1). The RBC diameters in the VSLT group had the second smallest value, being lower than those in the C, MST, and MSLT groups but statistically significant only when compared to the MSLT group. They were higher than those in the VST group, but this difference was not statistically significant. The RBC diameters in the MST group were measured for the cells that still displayed a round, regular shape. The obtained mean value was similar to that calculated for the C group (and close to the VSLT group), and it was also smaller than that of the MSLT group with no statistically significant differences in all cases. However, the RBC diameters in the MST group were statistically significantly higher than those in the VST group. The mean RBC diameter in the MSLT group was the highest; however, this difference was only statistically significant when compared to the values from the venom-treated groups.

### 2.3. Haematological Data

#### 2.3.1. Haematocrit

The haematocrit (Hct) data showed small differences among the five experimental groups, but these differences were not statistically significant. In all of the treatment groups, the haematocrit data were lower than those in the C group (ranging 41.08–43.78%), but these differences were not statistically significant, either. The mean value was for the VST group was lower than that in the melittin-treated groups and higher than that in the VSLT groups. The Hct value in the VSLT group was the smallest among all of the experimental groups. The haematocrit values in the MST group were the highest among all of the treatment groups, while the values in the MSLT group were lower than those in the MST group but higher than those in the venom-treated groups.

#### 2.3.2. RBC Number

The analysis of the RBCs revealed important differences among the five experimental groups with statistical significance in several cases (Table 2). The mean number of RBCs in the VST group was higher than those in the C, VSLT and MSLT groups (with statistical difference only when compared to the MSLT group) and lower (and not significant) than that in the MST group. As already discussed for the shapes, in the VST group, a defective haematopoiesis resulted in smaller and more numerous RBCs, which was confirmed by their reduced volume and Hct. The number of RBCs in the VSLT group was lower than those in the C, VST and MST groups (statistically significant difference only from the MST group), while it was significantly higher than that in the MSLT group (Table 3). The number of RBCs was the highest in the MST group and statistically significantly different than those in the VSLT and MSLT groups. Finaly, the mean number of RBCs in the MSLT group was statistically significantly lower than those calculated for all of the other experimental groups.

#### 2.3.3. RBCs Volume

There were also differences in the volume of the RBCs among the five experimental groups with statistical significance in several cases (Table 3). In the VST group, the RBC volume was lower than that in the C group and the lowest among all of the experimental groups (as for diameters); however, this finding was only statistically significant compared with the MSLT group. In the VSLT group, the RBs volume was non-significantly higher than those in the C, VST and MST groups, while it was significantly lower than that in the MSLT group. The RBC volume in the MST group was lower than those in the C group (without statistical significance) and MSLT group (statistically significant). The RBC volume in the MSLT group was statistically higher than those measured for all of the other groups.

#### 2.3.4. RBC Area

The analysis of the RBC surface area revealed a similar pattern to that for the diameters with important differences among the studied groups—some of which showed statistical significance (Table 4). The lowest value was recorded again in the VST group, but this difference was statistically significant only when compared to the values in the C and MSLT groups. The second lowest value was recorded for the VSLT group, but this was significantly lower only when compared to the MSLT group. The MST group had the third lowest surface area, but this difference was not statistically significant compared to the values from the C group or the other previously mentioned groups. However, this value was statistically lower than that of the MSLT group. The mean surface area of the RBCs in the MSLT group was the highest among all of the groups, and this finding, similarly to those for the number of RBCs and their volume, was statistically significant.

#### 2.3.5. RBC $\frac{V_{w}}{A}$ Ratio

The $\frac{V_{w}}{A}$ ratio was the highest in the VST group (0.39), which was followed by the values from the VSLT, C, and melittin-treated groups (with almost identical values, ranging 0.36–0.37), but these differences were not statistically significant. 

### 2.4. NMR Data

#### 2.4.1. Transverse Relaxation Time of the Water Proton from the Cell Interior

The calculated mean values for the transverse relaxation time of the water proton from the cell interior (T_2i_), at both temperatures, were the lowest in the VST group, but this finding was only statistically significant when compared to that of the MSLT group (Table 5). The T_2i_ values at 25 °C increased in the following order: C group, VSLT group, MST group and VSLT group, while at 37 °C, the values from the MST and VSLT groups were inversed (but close to each other). The measurements performed at 37 °C also revealed significant differences between the MSLT group and the C and VSLT groups. Upon further comparing the T_2i_ data recorded at the two temperatures, we noted statistically significantly increased values for all groups at 37 °C.

#### 2.4.2. Water Proton Relaxation Time

The calculated mean values for the water proton relaxation time (T_2a_), at both temperatures, were the lowest in the VSLT group, and they increased as follows: MST, VST, MSLT, and C. Statistically significant differences were found between the results of the VSLT and MST groups and those of the control group (both at 25 °C and when comparing the groups at different temperatures; see Table 6).

#### 2.4.3. Water Exchange Time

The mean water exchange time (T_e_) in the VST group showed the same characteristics as those for T_2a_, including all the statistically significant differences (Table 7).

#### 2.4.4. Diffusional Permeability for Water

Finally, the mean diffusional permeability (P_d_) of the RBCs was the lowest in the C group at both temperatures and increased in the following order: MSLT, MST, VST and VSLT groups; these differences were statistically significant between the VSLT and C groups (again at both temperatures). The differences were also statistically significant when comparing the results for the same groups at 25 °C and at 37 °C with higher values recorded at 37 °C (Table 8).

It is also important to mention that for all of the NMR parameters, statistically significant differences were observed between the results obtained at 37 °C and 25 °C: values for T_2i_ and P_d_ were higher, while those for T_2a_ and T_e_ were lower.

## 3. Discussion

Circulant mammalian RBCs remain the most important experimental model for testing the permeability of biological membranes. In this paper, this model was used to investigate the transport of water molecules across the plasma membranes, which was stimulated by the in vivo presence of BV molecules and Mlt alone. Many researchers have focused on the biophysical and biochemical effects of Mlt, BV or venoms produced by other species on membranes, including RBC membranes. Also, most of the studies in the literature compared the water permeability of the RBCs from various species without administering any experimental treatment. However, our results regarding the in vivo changes of the RBC membranes’ water permeability correlated with those of the RBCs’ morphology, and the haematological results are entirely original. The subcutaneously injected BV and Mlt diffused from the site of administration into the subjacent tissues and eventually arrived in the blood flow. This way, in both experimental conditions (subchronic and acute, semi-lethal treatment), the venom molecules and Mlt, respectively, came into contact with the circulant RBCs and produced important morphological and functional changes in the plasma membrane. This paper found statistically significant differences among the five experimental groups for almost all of the analysed parameters.

The shape of biconcave discs observed for the RBCs of rats in the C group is normal and specific to all mammalian RBCs [42,43]. The RBCs in all treatment groups suffered smaller or bigger changes in their shapes, which was dependent on the substance tested and on the experimental condition. An intriguing and original aspect revealed here is represented by the prevalence of a certain abnormal shape in the different groups. Thus, stomatocytes were most numerous in the subchronic treated groups (VST and MST), while acanthocytes were most numerous in the VSLT group, and echinocytes prevailed in the MSLT group. In all cases, the presence of some less damaged cells suggested either a continuous process of alteration or a differentiated resistance of the RBCs to the tested substances, which was probably due to their age.

The lower number of RBCs in the blood of the rats injected with cumulative BV doses was previously reported and explained as an important comeback after the initial extensive deleterious effect of the BV molecules on the circulating RBCs, which featured an essential contribution from the haematopoietic bone marrow [44,45,46]. A similar process was reported by Ginsberg et al. [47] after the administration of Mlt. In this paper, it is interesting that in both subchronic treatments (with BV and Mlt), stomatocytes prevailed as the most common form of abnormal cells. This suggests that the presence of low amounts of Mlt in the blood and in the bone marrow of the treated rats was the main factor responsible for the morphological changes that initiated during haematopoiesis. The other venom molecules were probably responsible only for minor changes.

In the VSLT group, almost all of the cells were acanthocytes, some of which turned into echinocytes, which was the prevalent form of abnormal RBCs in the MSLT group. In these cases, the high number of BV molecules, or Mlt, appeared in circulation and acted suddenly and directly on the RBC membranes. The differentiated effect on the cells from the same blood sample could be explained again by the various ages of the affected cells and their related varying levels of resistance against the BV molecules. The general toxic effect observed in the VSLT group compared to the MSLT group (where not all the cells were damaged) could be the result of the presence of high amounts of BV molecules, some of which potentially affected the RBC membrane. However, only phospholipase A_2_ (PLA_2_) produces relevant perturbations of the RBC membrane bilayer. This enzyme alone has low toxicity, but it is activated by high doses of melittin [48,49], which is when it becomes a major haemolytic factor [18,50,51]. The structure and effects of the BV PLA_2_ were recently analysed in a comprehensive review [52]. The more advanced changes found in the VSLT group compared to the VST group also indicate a dose-dependent effect. This was also observed in the two Mlt-treated groups, where the number of cells with aberrant shapes was much higher in the MSLT group compared to the MST group, and they also appeared to be larger.

Different abnormal RBC shapes, including those reported here, were correlated with pathological situations [53]. Our results are consistent with data reported by Tosteson et al., who crenated RBCs through in vitro incubation with Mlt [54], and by Hur et al., who analysed the in vitro dependence of the RBC shapes on the concentration of Mlt [55]. According to the latter study, our findings also suggest that the concentration of Mlt in the blood of the rats (even after the high injected doses) did not reach the level required to trigger the transformation of the RBCs to spherocytes. And, unlike all the in vitro studies, we found specific aberrant shapes in different experimental conditions. Sandesha et al. [30] reported crenated cells or serrated plasma membrane projections of the RBCs in relation with cytoskeletal changes when analysing envenomation by the *Hypnale hypnale* snake, which eventually resulted in the aggregation of washed cells. Furthermore, Yau et al. [41] observed an evolution of the morphological changes of RBCs in vitro in the presence of the snake *Pseudechis guttatus* venom from normal discocytes to stomatocytes and later to spherocytes, which was eventually followed by haemolysis. These authors also associated the cellular changes with some cytoskeletal rearrangements. This impact on the RBC membrane cytoskeleton could also be generated by Mlt or other BV molecules. This paper also demonstrated an in situ lytic effect of Mlt through the statistically significant lower number of RBCs recorded in the MSLT group compared with all the other groups and the C group in particular.

The RBC diameters in the C group showed a mean value slightly smaller than previously reported [56]. Also, the mean values reported for Hct, RBC number and volume in the C group are consistent with those available in the literature [56,57]. All haematological parameters had bigger or smaller changes in the treated groups, but the most important changes (and with higher amplitude) were found, as expected, in the BV-treated groups. Reduced RBC diameters were measured in these two groups. Compared with the C group, the VST group had a significantly lower mean diameter (by about 10%) as well as reduced Hct (not significant) and lower volume, while the VSLT group reported slightly larger values. In contrast, the MSLT group had significantly larger RBC diameters and reduced Hct levels compared to the C group. The results showed lower diameters and Hct after BV administration compared with Mlt administration. Compared to the MST group, in the VST group, lower values were found for the RBC diameters (by about 10%, which was statistically significant), Hct, number of RBCs, and cell volume. In the VSLT group, lower values (by about 9%) were found for the RBC diameters (which was statistically significant), Hct, and cell volume (by about 8.5%, which was statistically significant), while the number of RBCs was significantly higher than that in the MSLT group. When comparing the effects of the same substance in different testing conditions, BV subchronic injection resulted in lower diameters and volume and a higher number of RBCs than after its semi-lethal administration. Concerning the effects of Mlt, the MST group showed lower diameter and volume (which was statistically significant) alongside higher Hct and numbers of RBCs (by 12%, which was statistically significant) compared to the MSLT group.

These results show the distinct effects of BV and Mlt administration in different experimental conditions. On one hand, injections of low doses of BV over 30 days were more toxic to the RBCs than a single high dose. An explanation for the larger number could be a stimulation of haematopoiesis consecutive to the haemolysis produced in the first 1–2 weeks of treatment, as discussed above. The same distribution of the results was seen in the Mlt-treated groups, where lower diameters and volumes were recorded in the MST group. Also, a high number of RBCs was found in this group, even higher than in the VST group, suggesting that Mlt is the BV molecule responsible for the stimulation of haematopoiesis. A dose-dependent altered number of RBCs was also found by Shokhba et al. [58] consecutive to an acute in vivo envenomation of rats with high doses of *Naja nubiae* snake venom. The deleterious effect of the snake (viper) venom on the RBCs number was also time dependent [59].

The insertion of Mlt in the RBC membranes, following both subchronic and acute treatment either with the crystallised BV or with Mlt alone, resulted in changes in the diffusional water permeability of the RBCs. Studying the permeability of a membrane is a laborious method, but it also is the most precise and adequate. It was our intention not only to see whether water passes through melittin pores but also to calculate the effect of different in vivo experimental conditions on its passage. All the directly measured parameters are relevant only in the context of calculating the water permeability. The mean P_d_ values in the C group for the rat RBCs were much smaller than previously reported [60]. In all of the treated groups, the P_d_ values were higher than those in the C group. Two temperatures were selected for the measurements: the laboratory temperature and the physiological body temperature of the adult rat [57]. At 25 °C, the P_d_ values increased in the venom-treated groups by 14% (in the VST group) and 18.5% (in the VSLT group), respectively, while in the Mlt-treated groups, the mean values of P_d_ were 8% higher (in the MST group) and 4% higher (in the MSLT group) than those in the C group. When considering the treatments with the same substance, the P_d_ values were higher in the VSLT group than in the VST group, while in the MST group, the P_d_ value was only a little higher than that in the MSLT group. On the other hand, the water diffusional permeability of the RBCs from the VST group exceeded that of the MST group (by 3.5%); a similar trend was found in the semi-lethal-treated groups with P_d_ increasing by 2.5% in the VSLT group. The same pattern was also observed for all groups when analysing the P_d_ at 37 °C but with higher mean values and higher percentages. When comparing the data obtained at the two temperatures, the P_d_ mean values increased in all of groups: by 35% in the C group, 36% in the VSLT and MSLT groups, and 37% in the VST and MST groups. This indicates that the BV molecules induced a more elevated water diffusional permeability than Mlt alone in all of the experimental conditions.

These different P_d_ values result from the various amounts of molecules that diffused from the injection sites into the blood and the time during which they acted on the RBC membranes. Consecutive to the acute treatments, more BV molecules (including PLA_2_) attacked the RBC membranes and influenced their properties. During the subchronic treatment, Mlt molecules were inserted daily and stepwise in the membranes of circulant RBCs and remained there to generate increased P_d_ values, while other BV molecules had less influence on this process. And, for the same reason, the P_d_ value was higher in the BV-treated groups than in the Mlt-treated groups. It also remains possible that the long-lasting presence of BV molecules, mainly Mlt, in the blood could influence haematopoiesis, affect the RBCs’ precursors and result in RBC membranes with compromised permeability. On the other hand, the smaller P_d_ value in the MSLT group compared with the MST group could be explained by perturbations of the AQP1 conformation and function by the numerous Mlt molecules, which was consecutive to a disturbance of the normal arrangement of lipids, as discussed below. Despite their limited statistical significance, these results proved the ability of both BV and Mlt to increase the cells’ water permeability in vivo.

Even though the intermediate parameters measured or calculated varied somewhat among the experimental groups, the final results for the P_d_ values had a certain logical hierarchy. A possible explanation is that the water exchange times could depend on the shape, volume, or number of the RBCs. Then, different amounts of the active substances arrived at the RBCs; in addition, a relative variability regarding the individual response of the low number of tested animals to the treatments could have influenced the results.

The in vitro permeation of RBC membranes by Mlt was previously described [54,55], reporting rapid changes in the RBC shapes, which was quickly followed by haemolysis. Our results are in line with the literature data and confirmed that Mlt was the main BV molecule that, due to its amphipathic nature, was able to diffuse from the injection site into various body fluids and arrive at the RBCs, where it induced changes in their membrane permeability. This outcome, based on the physico-chemical properties of Mlt, was already demonstrated in vitro in various experiments using biological or artificial membranes. Mlt is an amphipathic molecule with 20 hydrophobic amino acids residues at the N-terminal segment and 6 highly cationic, hydrophilic amino acids at the C-terminal end [61]. It adopts a tetramer conformation when suspended in phosphate buffer [62], in other concentrated salt solutions [63], or consecutive to its insertion into membranes, when the α-helix content of the molecule also increases, which involves 24 amino acid residues [61]. Habermann described Mlt as a “direct haemolysin” due to it altering the RBC membranes’ selective permeability for water, ions and even glucose at very low concentrations (10^−6^ M) [18,21]. Working with liposomes, Ohki et al. proved that electrically neutral phosphatidylcholine membranes were more susceptible to perforation, the formation of vesicles, and micellisation at lower concentrations of Mlt compared to the negatively charged phosphatidylserine membranes [64]. Mlt is adsorbed on phosphatidylserines by electrostatic interactions and is therefore kept for a longer time at the bilayer surface [65]. Our results are in line with these reports, since phosphatidylcholines and sphingomyelins range as the most abundant phospholipids in the RBC membranes [66,67], explaining the ability of Mlt to permeabilise them. It is also known that cholesterol, which constitutes up to 40% of the RBC membrane lipids [67], reduces their sensitivity to Mlt [68,69]. This is why the insertion of Mlt in the RBC membranes was somehow limited in our study without leading to generalised haemolysis.

Currently, the literature describes two distinct mechanisms of Mlt interaction with phospholipid membranes, which have different results. The first one states that the random coiled Mlt monomers partially insert themselves perpendicularly in the membrane [70] with the hydrophobic N-terminus, which is also responsible for their haemolytic activity. Shortly after, the monomers arrange parallel to the membrane, in contact with the hydrophobic chains of the lipids, and associate into tetramers [71]. By insertion into a biological membrane, Mlt directly influences the mobility of lipids, and it significantly decreases their lateral diffusion rate [72]. The result of inserting a large number of Mlt molecules in membranes (arriving at a certain threshold concentration) is permeation by removing small lipid-protein aggregates covered by Mlt [73]. According to the second mechanism, when present in large amounts, Mlt takes a tetrameric helix conformation, perpendicular to the membrane, with a tendency to partially crossing the bilayer [73,74], since the Mlt molecule has a single polar region. These amphipathic helixes of Mlt will result in assembling pores with relatively selective permeability [75]. Berneche et al. found that residues Ile^17^-Ser^18^-Trp^19^ in the C-termini of Mlt molecules form a favourable region for the passing of water [76] through 2.5–3 nm pores in liposomes [77] or in the RBC membranes [78]. The predominance of one mechanism or the other is determined by several factors, including Mlt concentration in the environment, the phospholipid composition of membranes and their electrical charges, environmental temperature and pH, etc. And to further complicate these mechanisms, it was shown that Mlt was responsible for an in vitro dose-dependent aggregation of the intrinsic membrane proteins from the RBC membranes [79], modifying the density of AQPs and producing fluctuations in the lipid bilayer. Due to these specific interactions with cell membranes, Mlt was tested against different types of cells, the most relevant being its antitumoral activity [80,81,82,83,84,85]. We could not prove here that one mechanism or the other prevailed and resulted in the increased permeability for water (other explanations such as changes in cell shape, or ionic balance, should also be considered). However, our results bring new insights into the interactions of BV and particularly of Mlt with biological membranes and could be useful for explaining its effects and finding new medical applications of this biological peptide.

As a limitation of this study, we must mention that our results regarding the water permeability consecutive to the inhibition of AQP1 with (4-carboxyphenyl)-chloromercury (PCMB) were not always consistent within the same group, which was mainly due to the different degrees of haemolysis in some samples corroborated with extensive, total haemolysis in the Mlt-treated groups. Also, technical difficulties were encountered during the manipulation of the limited amount of blood available from some of the rats. Data related to AQP inhibition could bring additional insights to the involvement of Mlt in the transport of water across the RBC membrane. However, our recorded measurements suggest that apart from the actual inhibition of the AQPs, PCMB also inhibited somehow the transport of water through Mlt pores. Meanwhile, the mean inhibition of diffusional water permeability in the C group was 33%, while the inhibition of the water transport process in the VST group and VSLT groups reached 50% and 57%, respectively. In future research, an inhibitor of PLA_2_ could be used to eliminate the contribution of this enzyme to the water permeabilisation of RBC membranes.

## 4. Materials and Methods

### 4.1. Saline Buffer

A saline buffer (SB) with 150 mM NaCl, 5.5 mM glucose, and 5 mM HEPES [4-(2-hydroxyethyl)-1-piperazine-ethanesulfonic acid] at pH = 7.4 (measured with a Corning Pinnacle 542 pH-meter, from Corning Inc., Corning, NY, USA) was used to reconstitute the bee venom (BV) and melittin (Mlt) for the injectable solutions and wash the RBCs. The RBCs were suspended in SB supplemented with 0.5% bovine serum albumin (BSA) for light microscopy and nuclear magnetic resonance (NMR) data. 

### 4.2. Bee Venom and Melittin Solutions

BV was collected by A.F. from a local race of European honeybees (*Apis mellifera carpatica*) as previously described [86]. A BV Mlt molecule of 2846.46 Da was purchased in lyophilised form at 96% purity from Sigma-Aldrich (St. Louis, MO, USA), product number M2272, Lot#120M4067V. The lyophilised Mlt was stored in the original brown bottle at −20 °C. The pure, crystallised BV, as well as the BV and Mlt reconstituted in SB, were kept at 0–4 °C and away from light (also in brown bottles) until use. A Sartorius Research R2000D balance (Sartorius GmbH, Göttingen, Germany) was used for precisely weighing the BV and Mlt powders.

### 4.3. RBC Doping Solution

For the nuclear magnetic resonance measurements, a doping solution (DS) with 40 mM MnCl_2_ and 100 mM NaCl was prepared.

### 4.4. Animals and Treatments

Thirty male Wistar rats (*Ratus norvegicus*) provided by the animal house of “Iuliu Haţieganu” University of Medicine and Pharmacy, Cluj-Napoca, Romania were used in this study. The number of animals was restricted for ethical reasons, and the animal suffering was minimised. The rats weighing 148.78 ± 18.21 g were separated into five groups (*n *= 6/group) separately housed in cages of 40/25/20 cm with no restrictions on water and movement. The food (standard rat chow from Cantacuzino Institute, Bucharest, Romania) was limited to 10 g/animal/day to preserve a relatively constant body weight during the experimental treatments.

The rats in the first group were injected daily for 30 days with 100 μL of SB; this group was considered the control group (C group). Rats in the VST (venom subchronic-treated) group were injected daily for 30 days with a dose of 700 μg BV/kg/day (equivalent to the venom released by one bee sting [21]) to mimic a subchronic treatment. Rats in the VSLT (venom semi-lethal-treated) group were injected with a single dose of 62 mg BV/kg (equivalent to 100 bee stings), which was calculated by us as a median lethal dose, LD_50_ [86,87], to mimic an acute, semi-lethal treatment. Rats in the MST (Mlt subchronic-treated) group were injected daily for 30 days with a dose of 350 μg Mlt/kg/day (equivalent to one bee sting) to mimic subchronic treatment. Rats in the MSLT (Mlt semi-lethal-treated) group were injected with a single dose of 31 mg Mlt/kg, equivalent to 100 bee stings, to mimic semi-lethal treatment. The BV and Mlt solutions were subcutaneously injected in the dorsal, lumbar region in order to reproduce the way venom naturally spreads into the body. The volumes of BV and Mlt injected in very high doses were distributed across several injection sites to increase the rate of molecule absorption in circulation.

At the end of each experimental treatment (4 h after the small daily doses and 2 h after the high doses), the rats were generally anaesthetised in an atmosphere of chloroform, and blood samples were collected by cardiac puncture.

### 4.5. Morphometric Analyses

First, 100 μL of whole blood/rat was added within seconds of the sampling to ice-cold 3 mL 0.5% BSA in SB. Morphometric analyses of the RBCs were performed as previously described [88]. Then, 50 μL of blood cells suspension was placed on microscope glass slides and covered with cover glasses, and the suspension excess was removed with filter paper. The samples were studied in phase contrast with a Nikon Eclipse 80i light microscope (Nikon Corporation, Tokyo, Japan). More than 10 different fields were photographed for each group using an Olympus Color View 1 CCD camera (Olympus Soft Imaging Solutions GMBH, Münster, Germany). Afterwards, 100 to 300 RBCs/group were manually measured with a CellD Olympus software, version 3.1 (Olympus Soft Imaging Solutions GMBH, Münster, Germany). The diameters (D) of RBCs were expressed in μm.

### 4.6. Haematological Analyses

Subsequently, another 2 mL of whole blood/rat was transferred again within seconds into specimen BD-vacutainers pretreated with K_2_EDTA. These blood samples were subjected to haematological analyses on a Sysmex XT 1800i flow cytometric haematology analyser (Sysmex Co., Kobe, Japan). Haematocrit (Hct) and RBC counts (RBCs) were considered, and the mean obtained values were used to calculate the mean volume of RBCs (V), according to the following formula:

(1) $$V=\frac{Hct}{RBCs}\times 10$$

The RBC diameters (D) and volumes were used to calculate the RBCs’ surface area (A) according to the following formula:

(2) $$A=\frac{\pi \times D^{2}}{2}+\frac{4\times V}{D}$$

The obtained values for RBC volume (V) were also used to calculate the RBCs’ water volume (V_w_) according to the following formula:

(3) V_w_ = 0.7 × V

Then, the V_w_ and surface area (A) values were used for the calculation of the $\frac{V_{w}}{A}$ ratio, which was used to measure the water permeability.

### 4.7. RBC Permeability Assessed by NMR

Finally, approximately 6 mL of whole blood/rat was collected and transferred into other BD-vacutainers pretreated with K_2_EDTA (3 × 2 mL/rat). The RBCs were separated during three washes with SB separated by centrifugations for 10 min at 2000× *g* in a Jouan CR3i centrifuge (Jouan S.A., Herblain, France). Then, 600 μL of RBCs sediment/tube was further centrifugated for 60 min at 10,800× *g* in a Wifug X-1 centrifuge (Wifug, Stockholm, Sweeden) in order to completely eliminate the water among the RBCs in the samples. The resulted sediments, at an Hct of at least 95%, were used to measure the transverse relaxation time of the water proton from the cell interior (T_2i_), which was excited at 20 MHz without Mn^2+^ ions [89]. Afterwards, 7 different measurements were performed for the RBCs of each rat in all 5 experimental groups at both temperatures.

From the remaining sediment, 200 μL of RBCs was resuspended in 200 μL of 0.5% BSA in SB and 200 μL of DS. These RBCs were used to measure the water proton relaxation time (T_2a_), which was excited at 20 MHz and in the presence of Mn^2+^ paramagnetic ions. An additional 7 different measurements were performed for the RBCs of each rat in all 5 experimental groups at both temperatures.

T_2i_ and T_2a_ were measured with a Bruker Minispec ^1^H-NMR spectrometer and the dedicated Soft-EDM t2_cp_mb software, version 1.2 (Bruker Optik GmbH, Ettlingen, Germany), according to a method previously presented [90,91]. These parameters were measured at two different temperatures, and a Julabo F25 thermostat (Julabo Labortechnik GmbH, Seelbach, Germany) was used to bring the blood samples to the working temperatures of 25 °C and 37 °C, respectively.

The mean values of the 7 measurements of the relaxation times T_2a_ and T_2i_ obtained for each animal at either of the 2 temperatures were then used to calculate the water exchange time between the two sides of RBCs (T_e_), using the following formula [90]:

(4) $$\frac{1}{T_{e}}=\frac{1}{T_{2a}}-\frac{1}{T_{2i}}$$

The mean T_e_ values and the $\frac{V_{w}}{A}$ values previously calculated were finally used to calculate the water diffusional permeability (P_d_) of the rat RBC membranes at 25 °C and 37 °C, in the different experimental groups, using the following formula [91].

(5) $$P_{d}=\frac{V_{w}}{A}\times \frac{1}{T_{e}}$$

### 4.8. Statistical Analysis

The morphometric and haematological data, as well as the NMR results from the control group and for the different treated groups, were expressed as mean ± standard deviation. The values obtained for each parameter in the different groups were analysed with one-way ANOVA for multiple series of data (at *p* < 0.05 considered significant), and the results from different groups were compared using a post hoc Tukey’s test (at *p* < 0.05 considered significant). Origin 7.0 software, version SR1 (OriginLab Corporation, Northampton, MA, USA) was used to perform the statistical analysis.

## 5. Conclusions

The methodology used here enabled the analysis of the interactions of BV molecules, mainly Mlt, with the membranes of the RBCs. These cells represented a perfect experimental model, and the ^1^H-NMR spectroscopy was the most adequate tool for assessing the membrane permeability for water. This is the first study to precisely measure the in vivo effects of the BV molecules on this process.

The insertion of Mlt in the RBC membranes, following both the in vivo subchronic and acute treatment, either with crystallised BV or with Mlt alone, resulted in important morphological changes in all of the experimental conditions. Apart from the presence of cells with aberrant shapes (stomatocytes, acanthocytes or echinocytes—each form prevailing in certain groups), the other investigated parameters were significantly modified (RBC diameter, count, and volume). Even if the tested molecules were differentially absorbed and filtrated, they arrived at the RBC membranes in large amounts. In all of the treated groups, the P_d_ values were higher than those of the C group and increased with the temperature. The RBCs in the VSLT and VST groups had the highest mean values of P_d_ at 37 °C, which were followed by those calculated for the MST and MSLT groups and the C group. These results demonstrated the ability of Mlt to retain its ability to interact with the RBC membrane in vivo and confirmed that Mlt is the most important molecule of the BV involved in this process.

## Figures and Tables

**Table 1:** Statistical analysis of RBC diameters in the different experimental groups.

Experimental Groups	N	Average (μm)	SD	ANOVA
C group	6	6.3465	0.4253	*p* = 0.00026
VST group *^,1,2^	6	5.7442	0.1212	
VSLT group ^3^	6	6.2338	0.4276	
MST group ^1^	6	6.3592	0.3692	
MSLT group ^2,3^	6	6.8692	0.2891	

* Statistically significant difference compared to the C group (Tukey’s test).^1-1,2-2,3-3^ Statistically significant difference from each other (Tukey’s test).

**Table 2:** Statistical analysis of the number of RBCs in the different experimental groups.

Experimental Groups	N	Average (10^6^/mm^3^)	SD	ANOVA
C group	6	8.9683	0.4190	*p* = 0.00021
VST group ^1^	6	9.0550	0.2581	
VSLT group ^2,3^	6	8.3433	0.3922	
MST group ^3,4^	6	9.2383	0.4771	
MSLT group *^,1,2,4^	6	8.0100	0.6004	

* Statistically significant difference compared to the C group (Tukey’s test). ^1-1,2-2,3-3, 4-4^ Statistically significantly different from each other (Tukey’s test).

**Table 3:** Statistical analysis of RBC volume in the different experimental groups.

Experimental Groups	N	Average (µm^3^)	SD	ANOVA
C group	6	48.946	1.3474	*p* < 0.0001
VST group ^1^	6	46.6907	2.1166	
VSLT group ^2^	6	49.2582	2.4273	
MST group ^3^	6	47.4035	2.0873	
MSLT group *^,1,2,3^	6	53.8737	1.8449	

* Statistically significant difference compared to the C group (Tukey’s test). ^1-1,2-2,3-3^ Statistically significantly different from each other (Tukey’s test).

**Table 4:** Statistical analysis of RBC areas in the different experimental groups.

Experimental Groups	N	Average (μm^2^)	SD	ANOVA
C group	6	94.4110	7.0018	*p* < 0.0001
VST group *^,1^	6	84.3515	1.9660	
VSLT group ^2^	6	93.03467	5.4438	
MST group ^3^	6	93.5565	5.9623	
MSLT group *^,1,2,3^	6	105.5905	5.4677	

* Statistically significant difference compared to the C group (Tukey’s test). ^1-1,2-2,3-3^ Statistically significantly different from each other (Tukey’s test).

**Table 5:** Statistical analysis of T_2i_ at 25 °C and 37 °C in the different experimental groups.

Experimental Groups	N	Average (10^−3^ s)	SD	Average (10^−3^ s)	SD	Tukey’s Test
Temperature	25 °C			37 °C		
C group	6	126.07	0.800	152.11	2.131	*p* < 0.0001
VST group	6	124.06 ^1^	23.459	146.96 ^1^	17.624	*p* = 0.0181
VSLT group	6	135.07	3.309	164.6 ^2^	4.643	*p* < 0.0001
MST group	6	138.45	11.318	163.75	14.788	*p* = 0.0076
MSLT group	6	146.73 ^1^	7.772	171.62 *^,1,2^	8.179	*p* = 0.0003
ANOVA		*p* = 0.023 (3–4)		*p* = 0.00493		

* Statistically significant difference compared to the C group (Tukey’s test). ^1-1,2-2^ Statistically significant differences from each other in the same column (Tukey’s test).

**Table 6:** Statistical analysis of T_2a_ at 25 °C and 37 °C in the different experimental groups.

Experimental Groups	N	Average (10^−3^ s)	SD	Average (10^−3^ s)	SD	Tukey’s Test
Temperature	25 °C			37 °C		
C group	6	6.3877	0.4085	4.7196	0.4524	*p* < 0.0001
VST group	6	5.7513	0.2166	4.3231	0.1593	*p* < 0.0001
VSLT group	6	5.3336 *	0.2952	4.0535	0.2581	*p* < 0.0001
MST group	6	5.4573 *	0.3184	4.2345	0.3395	*p* < 0.0001
MSLT group	6	5.8468	0.6827	4.5173	0.6568	*p* = 0.0064
ANOVA		*p* = 0.00186		*p* = 0.0813		

* Statistically significant difference compared to the C group (Tukey’s test).

**Table 7:** Statistical analysis of T_e_ at 25 °C and at 37 °C in the different experimental groups.

Experimental Groups	N	Average (10^−3^ s)	SD	Average (10^−3^ s)	SD	Tukey’s Test
Temperature	25 °C			37 °C		
C group	6	6.7296	0.4550	4.8720	0.4821	*p* < 0.0001
VST group	6	6.0483	0.2341	4.4555	0.1635	*p* < 0.0001
VSLT group	6	5.5534 *	0.3174	4.1561	0.2694	*p* < 0.0001
MST group	6	5.6845 *	0.3574	4.3493	0.3655	*p* < 0.0001
MSLT group	6	6.0940	0.7436	4.6430	0.6957	*p* = 0.0058
ANOVA		*p* = 0.00152		*p* = 0.07842		

* Statistically significant difference compared to C the group (Tukey’s test).

**Table 8:** Statistical analysis of P_d_ at 25 °C and at 37 °C in the different experimental groups.

Experimental Groups	N	Average (cm × s^−1^ × 10^3^)	SD	Average (cm × s^−1^ × 10^3^)	SD	Tukey’s Test
Temperature	25 °C			37 °C		
C group	6	5.5458	0.4224	7.5087	0.5056	*p* < 0.0001
VST group	6	6.3281	0.2119	8.6986	0.2518	*p* < 0.0001
VSLT group	6	6.5753 *	0.5294	8.9571 *	0.6487	*p* < 0.0001
MST group	6	5.9867	0.8846	8.2225	0.8455	*p* < 0.0001
MSLT group	6	5.7611	0.8332	7.8596	1.2809	*p* = 0.0100
ANOVA		*p* = 0.0257		*p* = 0.02366		

* Statistically significant difference compared to the C group (Tukey’s test).

## Data Availability

The data presented in this study are available on request from the corresponding author.

## Abbreviations

The following abbreviations are used in this manuscript:

A	RBC surface area
AQP	aquaporin
BV	bee venom
C group	control group
D	RBC diameter
Hct	haematocrit
Mlt	melittin
MSLT group	Mlt semi-lethal-treated group (a single dose of 31 mg Mlt/kg)
MST group	Mlt subchronic-treated group (30 daily doses of 350 μg Mlt/kg/day)
NMR	nuclear magnetic resonance
P_d_	diffusional permeability for water
RBC	red blood cell
T_2a_	water proton relaxation time
T_2i_	transverse relaxation time of the water proton from the cell interior
T_e_	water exchange time
VSLT group	venom semi-lethal-treated group (a single dose of 62 mg BV/kg)
VST group	venom subchronic-treated group (30 daily doses of 700 μg BV/kg/day)
V_w_	RBC water volume
